# Modeling Platinum Sensitive and Resistant High-Grade Serous Ovarian Cancer: Development and Applications of Experimental Systems

**DOI:** 10.3389/fonc.2014.00081

**Published:** 2014-04-17

**Authors:** Paula Cunnea, Euan A. Stronach

**Affiliations:** ^1^Molecular Therapeutics Laboratory, Ovarian Cancer Action Research Centre, Institute of Reproductive and Developmental Biology, Department of Cancer and Surgery, Imperial College London, London, UK

**Keywords:** high-grade serous ovarian cancer, platinum sensitive, resistant, tumor heterogeneity

## Abstract

High-grade serous ovarian cancer remains the most common sub-type of ovarian cancer and, characterized by high degrees of genomic instability and heterogeneity, is typified by a transition from early response to acquired resistance to platinum-based chemotherapy. Conventional models for the study of ovarian cancer have been largely limited to a set of relatively poorly characterized immortalized cell lines and recent studies have called into question the validity of some of these as reliable models. Here, we review new approaches and models systems that take into account advances in our understanding of ovarian cancer biology and advances in the technology available for their generation and study. We discuss primary cell models, 2D, 3D, and organotypic models, and “paired” sample approaches that capture the evolution of chemotherapy failure within single cases. We also overview new methods for non-invasive collection of representative tumor material from blood samples. Adoption of such methods and models will improve the quality and clinical relevance of ovarian cancer research.

## Introduction

Ovarian cancer is currently the fourth leading cause of cancer deaths in women in the UK and the most common cause of gynecological cancer deaths, with approximately 4300 deaths from the disease in 2011 alone (http://www.cancerresearchuk.org/cancer-info/cancerstats/types/ovary/). The mortality rate for ovarian cancer is high as disease largely remains undetected, due to the vague nature of its symptoms and lack of reliable biomarkers, until patients finally present with high volume, disseminated disease. The current standard care for ovarian cancer involves cytoreductive surgery followed by combination chemotherapy with platinum compounds and taxanes. However, chemoresistant disease typically recurs in patients, most commonly in the high-grade serous (HGS) sub-type, with a low 5-year average survival rate of less than 40%. Ovarian cancer is a very heterogeneous disease, with the four most common sub-types of epithelial ovarian cancer (EOC) being serous, endometrioid, mucinous, and clear cell. They can be further divided into low-grade type I (relatively resistant to platinum-based chemotherapy) and the more common high-grade type II (more responsive to initial platinum-based chemotherapy but paradoxically poorer prognosis) tumors. Type I tumors, including low-grade serous and endometrioid, mucinous and clear cell histotypes, make up 10–20% of EOC, present at early stage (FIGO I-II), genetically have near normal gene copy number, are usually wild type for p53 and harbor characteristic mutations in genes such as *Ras* (mucinous and low-grade serous) and *PTEN* (endometrioid) among others. Type II lesions including HGS cancers, undifferentiated cancers, carcinosarcomas, and high-grade endometrioid, typically present at advanced stage (FIGO III–IV), are characterized by high genomic instability (near 50% deficiency in Homologous Recombination repair), near 100% p53 mutation rate, and have extensive DNA copy number changes (1).

The development of platinum-resistant disease is a critical and poorly understood problem in ovarian cancer, especially in the most prevalent HGS sub-type. Broadly, two potential models for the evolution of chemoresistance in HGSOC are proposed; one suggests that treatment with DNA-damaging platinum therapy causes mutations that give rise to resistance and the other suggests that genetically heterogeneous tumor clones exist prior to chemotherapy and subsequent treatment preferentially selects resistant clones for survival while platinum sensitive clones are eradicated by chemotherapy treatment (2). A genomic analysis of cell lines derived from three serous ovarian cancer patients, both before and after acquisition of clinical platinum resistance, indicated that in addition to shared genomic features, sensitive and resistant tumor cells from the same patient also exhibit mutually exclusive genomic characteristics, indicating that rather than a direct linear evolution of resistance from sensitive disease in response to platinum challenge, platinum-resistant clones are present from the outset within the sensitive presenting tumor (3). However, the bulk of research in this area has suffered due to the lack of appropriate models for developing effective therapeutic solutions to counter chemoresistance; and an inadequate sampling of tumor tissue, potentially missing the rich heterogeneity of HGS disease and hence the ability to study its underlying biology. Furthermore, many mechanistic studies investigating platinum resistance have to date relied on cell lines in which platinum resistance is derived *in vitro*, the mechanisms of which may have little or no relevance in the clinical setting (4).

Therefore, there is an urgent need to develop new models of platinum-resistant and refractory ovarian cancer to help improve outcomes for patients with chemoresistant disease. In this review, we will outline the procedures, technical challenges, and applications of modeling platinum-based chemoresistance in primary tumor cell cultures derived from ascites and solid tumors; the development of new immortalized cell lines and currently available cell line models of platinum-sensitive and -resistant HGS; and alternative systems of capturing tumor biology and heterogeneity in HGS disease.

## Development of Primary Models of HGS Disease

The clinical relevance of cell line models is a topic that is often debated. The use of established cell lines, while certainly not without their merits, may misrepresent responses to targeted therapies and users should research carefully the nature of the cell line models they chose, and how closely they relate to the clinical condition. To uncover the molecular mechanisms driving EOC development and treatment, suitable disease models must be available to faithfully mirror the disease *in vitro* and *in vivo*. For the study of drug resistance, especially when testing novel therapies, *ex vivo* models or cell line models that closely mimic the *in vivo* situation are required. The use of patient material such as ascites (a rich source of tumor cells) or solid tumor allows us to derive primary tumor cell cultures that closely resemble the patient situation, therefore representing a more experimentally accurate model than poorly characterized immortalized cell lines, often of uncertain origin. Primary tumor cell cultures are kept for a relatively short period of time and can be cumbersome to start and maintain in culture, but can be developed into well-annotated secondary immortalized cultures. Different methodologies have been developed for the isolation of EOC tumor cells from ascites and solid tumors. Here, we outline a number of recently published methods to retrieve and culture EOC tumor cells, the methods for development of immortalized cell lines from primary cultures and options available for 3D cell culture systems that attempt to more closely model the *in vivo* setting.

### Isolation and culture of EOC tumor cells from ascites

Ascites fluid can be a rich source of tumor cells that are highly accessible following paracentesis from the patient. Isolation and primary culturing of tumor cells from ascites have become more widespread and several different methods have been established to achieve this aim (5–8). A widely used protocol for the propagation of EOC tumor cells was developed by Langdon et al. (6, 9). Freshly drained ascites fluid, mixed with heparin to prevent cell aggregation, is pelleted by centrifugation, resuspended in PBS, and subjected to gradient centrifugation using histopaque or Ficoll-hypaque to remove any contaminating erythrocytes. The resulting interface layer is washed in PBS prior to culturing in appropriate tissue culture media, monitored carefully for fibroblast or mesothelial cell contamination, with the EOC tumor cells sub-cultured upon confluency (6, 9, 10). Alternatively, Shepherd et al. mix ascites 1:1 with M199/MCDB105 growth medium and monitor EOC tumor cell growth in culture, relying on EOC cells adhering to the plastic and contaminating erythrocytes being removed in the first set of media changes approximately 4 days after initial seeding (7). Similarly, methodology favored by Mes-Masson and colleagues directly mixes the EOC cells with growth media with minimal manipulation of the ascites-derived EOC cells (11–14). The different adherence rates of particular cells can also be used to separate EOC tumor cells from ascites (15). In this study, ascites cells were seeded onto low attachment plates for 24 h, following which two distinct populations of cells were observed: multicellular aggregates floating in media; and spindle-like fibroblast cells adhered to the low attachment plates. Further characterization identified the non-adherent cell population to be epithelial cell adhesion molecule (EpCAM) and CA125 positive EOC tumor cells (15), thus indicating that differential rates of adherence to plastic can be used to minimize contamination from other ascites cell types.

Different media can also influence the growth of primary tumor cells. Originally optimized for the growth of primary ovarian surface epithelial (OSE) cells (16), a combination of M199/MCDB105 media has been used for culturing primary ovarian tumor cells derived from ascites or solid tumor (7, 11). Supplements such as EGF and hydrocortisone have also been used in different cell culture media preparations; however, use of these agents in culturing OSE cells have been shown to initiate EMT (17). Continuous growth of primary ovarian tumor cells can also be achieved using commonly used culture media RPMI or DMEM (9). Addition of pre-cleared autologous ascites fluid to EOC cultures may aid in enhanced growth of cultures (9). Successful establishment of ovarian serous carcinoma cell lines has also occurred using serum-free culture media (18, 19).

A significant problem that arises however in the growth of EOC tumor cells following isolation from ascites is the presence of other contaminating cells such as fibroblasts or mesothelial cells. Careful monitoring of tumor cells in culture from any culturing technique is required to prevent contamination of the cultures from these cell types. Proliferation of fibroblasts will cease after a number of passages; however, if not contained from initial culturing, fibroblasts can outgrow the tumor cells and take over the culture. Selective trypsinization with low concentrations of trypsin can be used to remove contaminating fibroblasts or mesothelial cells from primary cultures, as they tend to detach more rapidly from plastic than tumor cells (6). However, this may need to be repeated several times to maintain a fibroblast-free culture.

Recent advances have lead to the use of magnetic enrichment for detection or purification of cells from fluid specimens (20). The EpCAM (CD326) is a transmembrane glycoprotein expressed by particular epithelial cell types in healthy individuals; however, it is over-expressed in most carcinomas (21) and is a target in antibody-based therapies, e.g., trifunctional bispecific antibody catumaxomab was approved by the European Medicines Agency 2009 for the treatment of malignant ascites (21–23). The over-expression of EpCAM in carcinomas has been exploited in the development of purification systems such as CD326 microbeads (from Milteny Biotec, Germany) or BerEP4 Dynabeads (Life Technologies, USA) allowing for the enrichment or depletion of EpCAM positive cell populations from fluid specimens such as ascites or pleural effusions. An advantage to selectively enriching EOC tumor cell populations prior to culturing is reduced contamination from fibroblasts and other cell types in the initial culture, thus not requiring monitoring for overgrowth of contaminating cell populations. Immunofluorescent staining for EpCAM performed on ascites cells (pre-cleared using histopaque-gradient centrifugation) before and after EpCAM microbead purification can be used to confirm highly enriched EpCAM positive EOC tumor cell populations post purification (authors unpublished observations). However, when using this additional step for isolation of EpCAM positive epithelial tumor cells, one must take into account that tumor cells that do not express EpCAM or express EpCAM at very low levels will be excluded, thus potentially losing important sub-clones of tumor cells. Cells that have undergone EMT, such as circulating tumor cells (CTCs), may have low or absent levels of EpCAM so using such enrichment techniques may fail to isolate these cell populations (24).

### Dissociation of EOC tumor cells from solid tumors

Several laboratories over the previous decades have developed various methods for EOC tumor cell isolation from solid tumor and metastatic deposits. Langdon et al. have advocated mechanical dissociation of tumor fragments using crossed scalpels, with cell suspensions filtered through sterile gauze to remove any remaining cell clumps before placing in culture (6, 10). The laboratories of Nachtigal and Mes-Masson have employed both mechanical disruption of tumor tissue using cell scrapers and enzymatic disruption of tumor using collagenase and concluded that mechanical dissociation was the more efficient method for their purposes (13, 14). More recently, Sueblinvong and colleagues compared a number of enzymes (collagenase A, hyaluronidase, and dispase II) commonly used for tumor dissociation and digestion times to mechanical disruption, examining viability and proliferation of the isolated tumor cells, and determined that 30 minute incubation with dispase II was optimal for dissociation of viable EOC tumor cells for primary culture and downstream applications (25). As with tumor cell cultures derived from ascites, careful monitoring of tumor-derived EOC cells in culture is required to minimize fibroblast contamination.

### Development of immortalized secondary cell lines

Primary cell lines have the disadvantage that they are short lived and may only be sub-cultured for days, weeks, or at most a number of months. A secondary immortalized cell line can be obtained from a primary culture: these have the advantages in that one is studying a pure and expandable population of tumor cells, uncontaminated with fibroblasts or other stromal cells, and are a continuous source to be accessed repeatedly *ad infinitum*. Establishment of a secondary culture can be achieved either by spontaneous or induced transformation of cells, e.g., SV40 T antigen induced immortalization. Spontaneous immortalization of cells can occur as cells maintained in culture over time can overcome senescence without the addition of exogenous agents to induce immortalization (26). Recently, platinum sensitive and resistant HGSOC lines were derived by Letourneau et al. and deemed to be immortalized when passaged more than 50 times (13). Alternatively, cell line models, such as the cisplatin-resistant HEY ovarian cancer cell line, have been developed from xenografted ovarian tumors passaged in immunologically deprived mice (27).

Normal controls of cancer tissues for comparative studies are also required but spontaneous immortalization of cultured normal cells is extremely rare and in the case of normal breast cells has only been observed in epithelial cells (28). *In vitro* immortalization is therefore necessary to induce secondary cultures of normal cells. Different methods have been employed to overcome the growth arrest barrier including transduction of viral oncogenes [reviewed in Ref. (28, 29)], radiation treatment (30), or carcinogenic chemical treatment (31, 32): viral oncogenic transformation methods have been used most commonly and successfully. Immortalization of normal human OSE cells has been induced using various methods, for example, using telomerase and temperature-sensitive SV40 large T antigen (33) and more recently immortalization of fallopian tube secretory epithelial cells (FTSEC) have been established by expressing human telomerase reverse transcriptase and perturbing the p53 and pRb tumor suppressor pathways (34). However, a caveat to the establishment of any secondary culture is that the cell clones that survive and become immortalized may be derived from a subpopulation particularly well adapted to cell culture conditions, but may not necessarily be the best representation of the actual tumor.

### 3D model systems of HGS disease

The vast majority of data produced on ovarian cancer and therapeutic responses is based on 2D cell culture models, whether they are *ex vivo* primary cells in short-term culture or immortalized cell lines, which both have distinct advantages but ultimately do not represent the three-dimensional nature of the human *in vivo* situation. Within the peritoneal cavity, transformed epithelial tumor cells can freely disseminate and be carried by the flow of peritoneal fluid (35). Spheroids of tumor cells can adhere to peritoneal mesothelial cells, anchor in the submesothelial matrix, and invade to form secondary lesions (36, 37). Thus, mimicking this system of adhesion, migration, and invasion *in vitro*, and thereby creating a more physiologically relevant microenvironment, could improve the concordance between predictions made in the laboratory and the clinical situation.

The 3D cultures can be created using several different methods; culturing cells within extracellular matrix gels (38), on low-adherent plastics (15) or non-adherent (polyHEMA)-coated tissue culture plastics (39); hanging-drop culture methods (13, 40, 41); spinner flasks (42); or rotary cell culture system (39). Recreating the 3D architecture of tissues and solid tumors using these methods better recapitulates primary tumor architecture than 2D monolayer culturing of cells. The 3D system could also be used as a predictive preclinical model, treating tumor cells in this *ex vivo* environment and determining response to therapy. Several studies have emerged recently using 3D models to elucidate mechanisms of drug resistance in EOC (43–47). A large-scale study using 3D models of 31 epithelial cell lines, compared their biological and molecular features with 2D cultures, and determined their response rates to chemotherapy agents, with 3D cultures differentially expressing adherens junction proteins (47), and in concordance with previous studies, 3D cultures were frequently more chemoresistant than their 2D counterparts (43–46). Furthermore, gene expression profiles of 3D cultured cells differ significantly from their 2D profiles, with 3D cultures resembling more closely the tissue of origin (41, 48, 49). However, one study revealed no major gene expression profile differences in OVCAR-5 cells grown in either 2D or 3D culture (50). The differences between these studies may be due to cell line variability or variability in 3D systems used.

Kenny et al. among other groups, have taken the 3D model a step further and describe a 3D organotypic model of ovarian cancer metastasis, mimicking human peritoneum and omentum (35, 51). This model has the potential to advance our understanding of invasion and metastasis, allowing researchers to work with a highly physiologically relevant model. The 3D model can be assembled to histologically mimic the *in vivo* situation, with primary human omental mesothelial cells, primary human omental fibroblasts, and primary ovarian tumor cells to create patient-specific biology and drug treatment options *ex vivo* (35). As with all model systems, there are advantages and disadvantages: the main disadvantages being that the primary cultures are usually viable for only a short period of time (around 1 week) and that the 3D models lack vasculature, host immune cells, and other *in vivo* factors. However, an *in vitro* 3D model of EOC represents a significantly more complex experimental system than monolayer cell cultures for analysis of tumorigenesis and development of new therapeutic approaches.

In the next section, we will discuss the most relevant current cell line models of platinum sensitivity and resistance in HGS and how their development has aided our understanding of chemoresistance.

### Current cell line models of platinum-sensitive and -resistant HGS cancer

Several cell lines exist representing platinum-sensitive or -resistant HGS cancers, derived from patient tumor or ascites prior to chemotherapy/chemoresistance and following resistant relapse. The best known of these are three sets of platinum sensitive and clinically acquired platinum-resistant HGS cell lines established by Langdon et al. (10). These cell lines were derived from the ascites or pleural effusions of platinum-sensitive patients and again following their relapse with platinum-resistant disease. These were the first sets of clinically acquired sensitive and resistant HGSOC models and have become an important resource in the study of chemoresponse and resistance EOC (52–55). In two of the sets, ascites or pleural effusion was obtained prior to chemotherapy (PEO14 and PEA1), with the other lines derived from cells obtained either after chemotherapy (PEO1, PEO4, PEO6, PEA2, and PEO23) or radiotherapy (PEO16). PEO1 cells, while derived following chemotherapy treatment, were done so following chemosensitive relapse. Disappointingly, there have been few paired cell line models that accurately depict acquired resistance to chemotherapy. The laboratories of Mes-Masson and colleagues have also established similar cell lines representing platinum sensitive and clinically acquired resistance from the same patient and have also generated further unpaired platinum-sensitive and -resistant cell lines (13, 56). Table 1 shows the list of available paired HGSOC cell lines that exist currently.

**Table 1:** **Clinical characteristics of paired platinum sensitive and resistant HGSOC cell lines**.

Cell line nomenclature	Histology at diagnosis	Treatment course	Reference
PEO1/4/6	Poorly differentiated serous adenocarcinoma	*Cis*-platinum/5-fluorouracil/chlorambucil	(10)
PEO14/23	Well-differentiated serous adenocarcinoma	*Cis-*platinum/chlorambucil	(10)
PEA1/2	Poorly differentiated serous adenocarcinoma	*Cis-*platinum/prednimustine	(10)
OV2295/OV2295(R2)/TOV2295(R)	Serous adenocarcinoma	Cisplatin/topotecan	(13)
		Paclitaxel/carboplatin	
		Doxorubicin	

Cell line models of platinum sensitive and clinically acquired platinum-resistant HGSOC have been used over the past decade examine the hypotheses regarding clonal evolution of tumor heterogeneity and treatment failure in HGS cancer, and to develop novel therapies to reverse resistance. Cooke et al. used multiplex fluorescent *in situ* hybridization and array CGH profiling to characterize the Langdon et al. (10) cell line pairs of platinum-sensitive and -resistant HGS disease and determined, in these three cases, that platinum-resistant disease did not appear to evolve linearly from sensitive disease (3). Rather, their data implied that both cell lines shared a common ancestor from an earlier stage in tumor development. Due to the extent and type of genomic alterations observed between the pairs, they proposed that platinum-resistant disease arose from pre-existing resistant sub-clones that were selected for during chemotherapy treatment (3). Subsequent research in our laboratory using these paired HGS cell lines identified key drivers of chemoresistance including DNA-PKcs-mediated activation of pro-survival AKT following treatment with DNA-damaging platinum-based chemotherapy (55) and HDAC4-regulated STAT1 deacetylation and activation following platinum treatment of resistant cells but not sensitive ones (54).

*In vitro* platinum sensitive and resistant cell lines, derived artificially in the laboratory by continuous or regular repeated exposure to increasing concentrations of platinum drugs, are widely used to uncover and characterize drug-resistant mechanisms (57, 58). While they have the advantages of being well-established, easy to work with and have identified useful tumor biology, we suggest that due to the non-physiological manner in which resistance is created, they should not be used as a definitive model of platinum resistance for clinical research. We performed a gene expression profiling of clinically acquired resistance versus *in vitro* derived resistance, in cell lines derived from the same patient. This analysis showed very poor concordance in gene expression profiles between the two models (54). Many other unpaired, clinically acquired platinum-sensitive or -resistant EOC cell lines are available, and have been extensively studied but as is the limitation with immortalized cell line models, which inherently develop phenotypic and genotypic alterations over time due to prolonged passaging, many established cell lines do not adequately model the clinical condition they are intended to represent (59). Commonly used epithelial ovarian cell line models such as the platinum-resistant SKOV3 cell line and A2780 have come under the spotlight recently, with multiple studies suggesting that they are poor models of HGSOC (60, 61) as, at the molecular level, they do not closely resemble typical HGS tumors. In a separate study examining drug sensitivity in 3D cultures, SKOV3 lines were shown to have hallmarks of clear cell histology (47). However, limited as models of HGSOC, these cell lines do have a utility as general models of ovarian cancer, for example SKOV3 is a good model of AKT-driven ovarian cancer, harboring an activating point mutation in PIK3CA (61). Significant strides to re-characterize existing cell lines to allow informed experimental design and interpretation of data have been made in recent years. For instance, a panel of 32 reported ovarian cancer cell lines has been systematically classified into their correct histotypes with the aim of definitively identifying more reliable models of clear cell ovarian cancer (60). These recent studies should be taken into account when choosing an ovarian cancer cell line as a model system.

It is clear that new initiatives are required to generate well-annotated and -controlled models of HGS for the ovarian cancer research community.

## Alternative Models of Identifying Tumor Cell Heterogeneity in HGS Disease

### Circulating tumor cells

The identification of significant tumor heterogeneity in HGS has raised questions over how representative single biopsy sampling of tumor material is. Alternative and/or complementary methods and models for tumor identification and monitoring of tumor burden are required. Over the last decade, the need for alternative models has seen a focus on CTCs and the isolation and analysis of CTCs in peripheral blood as a model of tumor characterization and evolution. First detected in 1869 by Ashworth in the blood of a patient with metastatic cancer, CTCs have been implicated in the development of distant metastasis (62). Furthermore, the potential prognostic role of CTCs has been demonstrated with the number of CTCs at any given time in peripheral blood, in certain tumor types, e.g., breast (63), lung (64), and prostate (65), predictive of disease progression, thus allowing for monitoring of disease burden during therapy. Several different approaches have emerged to isolate and identify CTCs, for example microfluidics systems for detection of cytokeratin positive or negative CTCs (66) or EpCAM positive CTCs (67–69), or PCR-based methods for monitoring a panel of predefined genes in ovarian cancer (70, 71). However, there are a few key drawbacks to the detection of CTCs in peripheral blood. First, the low number of CTCs present in circulation makes the initial detection of tumor cells problematic. Second, the various methodologies employed to detect CTCs may not be identifying all CTCs in circulation depending on the experimental approach. Methods using EpCAM as the tumor cell selection marker will not detect sub-populations of CTCs that have undergone EMT or lost EpCAM expression by other mechanisms. Additionally, many CTC isolation methods use two-layer density-gradient centrifugation, thus leading to potential isolation of peripheral blood mononuclear cells in addition to CTCs. Altered immune profiles of mononuclear cells could bias PCR-based profiling of gene expression panels (72, 73). A recent study investigating the predictive value of CTCs in newly diagnosed and recurrent ovarian cancer patients was inconclusive, showing no correlation with clinical characteristics or patient outcomes (69), suggesting that more work is required to delineate the prognostic value of CTCs in ovarian cancer while establishing a robust system for CTC isolation. The attractiveness of the use of CTCs as a form of “liquid biopsy” to establish a diagnosis and to monitor cancer burden in patients undergoing therapy and thus a model of disease progression or resistance must be carefully considered. Advances in methodology for reliable isolation of CTCs represent a vital area for progress.

### Circulating tumor DNA

The last 2 years have seen the emergence of detection of circulating tumor DNA in plasma as a method of tracking the genomic evolution of the tumor in response to therapy (74, 75). Ease of processing and accessibility to samples makes this non-invasive system an enticing prospect for detection of disease, monitoring of tumor burden, and determining evolution of clonal heterogeneity. Whole genome, exome, and targeted deep sequencing of plasma tumor DNA as single or serial samples have demonstrated the validity of this system (76, 77). In particular, the recent study by Murtaza et al. tracked six patients with advanced breast, ovarian, or lung cancer over 1–2 years. They performed exome sequencing on multiple samples collected at different time points and observed changes in copy number (both gains and losses) and gene-specific mutations between samples. The somatic mutations found in plasma prior to and after each treatment course were analyzed to identify changes in mutation profiles that could be attributed to disease progression and drug resistance (78). A further potential advantage of this method, as with CTCs, is that it reduces samples bias that may exist using single-site biopsy sampling as ctDNA is more likely to represent the tumor genome from multiple tumor sites, thus reducing the emphasis of future analyses on single sub-clones that may not represent the most common tumor genome.

Such techniques for detecting CTCs and sequencing of ctDNA from liquid biopsies (blood and plasma) are expected to become commonplace and to be developed and validated for prognostication and patient stratification in future clinical trials without the need for invasive diagnostic procedures.

## Conclusion

It is becoming increasingly evident that in the study of cancer cells and in particular examining drug resistance in HGSOC, more *ex vivo* and relevant *in vitro* models must be developed that more closely resemble the *in vivo* tumor environment, an opinion shared by others in recent reviews (59, 79). To that end, one of the goals of the European OCTIPS (Ovarian Cancer Therapy – Innovative Models Prolong Survival) consortium is to establish new, paired HGSOC cell lines derived from patients who are platinum sensitive at presentation but subsequently relapse, in an effort to delineate the mechanisms behind the development of relapse and platinum resistance in HGSOC and furthermore to develop new *in vivo* systems such as the relatively high throughput avian chorioallantoic membrane (CAM) models (80) and patient-derived xenografts (PDXs) (81), to advance novel therapies to combat chemoresistance. A recent study highlighted the utility of the PDX model in HGSOC, as platinum response in the PDXs echoed clinical outcome (82), whereas Lokman et al. highlighted advantages of the CAM system in cost, throughput, and reproducibility, compared to mice, as an *in vivo* model for studying complex phenotypes in ovarian cancer (83). Ideally, a consensus needs to be reached on standardized protocols for the isolation of tumor cells from both ascites and solid tumor or metastatic deposits, both in platinum-sensitive and -resistant disease, and a standardized set of markers that will definitively differentiate EOC tumor cells from contaminating stromal cells, such as cancer-associated fibroblasts. Such efforts would ensure that the subsequent results and conclusions drawn from experiments performed on these primary tumor cell populations and subsequent immortalized tumor cells can be confidently and correctly interpreted.

## Conflict of Interest Statement

The authors declare that the research was conducted in the absence of any commercial or financial relationships that could be construed as a potential conflict of interest.
